# Impaired Vibratory and Reciprocal Inhibition in Soleus H-Reflex Testing in Children With Spastic Cerebral Palsy

**DOI:** 10.7759/cureus.55541

**Published:** 2024-03-05

**Authors:** Sangeeta Gupta, Abhimanyu Vasudeva, Gaurav Gupta

**Affiliations:** 1 Physiology, All India Institute of Medical Sciences, Gorakhpur, Gorakhpur, IND; 2 Physical Medicine & Rehabilitation, All India Institute of Medical Sciences, Gorakhpur, Gorakhpur, IND; 3 General Surgery, All India Institute of Medical Sciences, Gorakhpur, Gorakhpur, IND

**Keywords:** vibration, spasticity, reciprocal inhibition, hmax/mmax, hmax, h-response, h-reflex, dorsiflexion, cerebral palsy

## Abstract

Introduction

Cerebral palsy (CP) is a neurodevelopmental condition that results from an injury to a developing brain. Children with CP fail to execute precise, well-coordinated movements, and excessive muscular co-contraction or co-activation is a prominent attribute of CP. The normal reciprocal relationship between agonists and antagonists during voluntary movements is altered in patients with CP. H-reflex, which is often regarded as the electrical equivalent of the spinal stretch reflex, can be used to examine the overall reﬂex arc, including the Ia sensory aﬀerent strength and the spinal motoneuron excitability state. Furthermore, neuromodulatory influence of vibration on H-reflex has been found, which has been increasingly investigated to ascertain its potential use as an intervention in patients with increased spinal reflex excitability. Our goal was to identify the brain mechanism underlying the motor deficits by studying Soleus H-reflex changes during voluntary movement (dorsiflexion) and also to determine the role of vibration in H-reflex modulation in children with spastic CP.

Methods

Soleus H-reflex was recorded in 12 children with spastic CP (10-16 years) and 15 age-matched controls. Recordings were obtained at rest, during dorsiflexion, and during vibratory stimulation for each subject. H-responses (Hmax amplitudes and Hmax-to-Mmax ratio) were compared among the controls and the cases (CP), for the experiments performed, by the Wilcoxon signed-rank test. The recruitment curves depicting the distribution of mean H-response amplitudes with stimulus intensity increment, for dorsiflexion and vibration were compared among controls and cases by the two-sample Kolmogorov-Smirnov (KS) test. p-value <0.05 was considered as statistically significant.

Results

Hmax amplitudes and the Hmax-to-Mmax ratio increased (15 % and 12.2 % increment, respectively) from the resting values in the children with CP (p<0.05), while controls exhibited a decrease (reduction of 62% and 57 %, respectively) during dorsiflexion (p<0.05). Vibratory stimulation produced a decreasing trend in H-response measures in both the groups. There was about 15 % and 16 % reduction respectively among children with CP while that of 24 % and 21 % respectively among the controls. The differences in the recruitment curves (distribution of average H-response amplitudes with stimulation intensity) recorded during dorsiflexion and vibration experiments among controls compared with those with CP were found to be statistically significant by the two-sample KS test (p<0.0001).

Conclusion

The failure of H-reflex suppression during voluntary antagonist muscle activation suggests the presence of impaired reciprocal inhibition in spastic CP. The relatively modest H-response reduction caused by vibratory stimulation in children with CP provides limited evidence of vibratory regulation of the H-reflex in CP. More research into the mechanisms driving motor abnormalities in children with CP is needed, which could aid in therapy planning.

## Introduction

Cerebral palsy (CP) is the most debilitating and perpetual outcome caused by an early insult to the developing brain which manifests as a consequence of neonatal encephalopathy. It is the most prevalent cause of childhood physical disability [1]. The prevalence of CP in India is nearly similar to global estimates. An Indian systematic review and meta-analysis suggest an overall pooled prevalence of 2.95 per thousand children [2]. Although CP patients do not have progressive brain damage, there are long-term cascading impacts on overall function. Children with CP fail to execute precise, well-coordinated movements. Excessive muscular co-contraction or co-activation is a hallmark of the disordered gait [3,4].

While numerous studies indicate that children with CP exhibit weakness, the exact mechanism underlying this weakness is not fully understood, and it most likely stems from multiple factors [5,6]. Inability to activate agonist muscles voluntarily, alteration in contractile and non-contractile muscle morphology, or excessive antagonist muscle co-contraction or co-activation, which in turn reduces force or torque-generating capacity, are some proposed explanations [6-10]. Increased co-contraction or co-activity to varying extents has been found in selected muscles in participants with CP compared with those in healthy participants [5,6,8]. In addition to being a potential cause of weakness, a decreased capacity to activate targeted muscles and an increased propensity for antagonist muscles to co-contraction or co-activation represent the abnormal motor control features of CP and have been linked to compromised corticospinal activity and potentially compromised intraspinal pathways. However, the underlying brain processes attributable to the sustained coactivation pattern in people with CP remain largely unknown. One of the mechanisms known to be important for the coordination of antagonist muscles is Ia reciprocal inhibition. Ia reciprocal inhibition involves a group of interneurons, which are activated through collaterals from descending pathways in parallel with agonist motoneurons and project to antagonist motoneurons [11]. This normal reciprocal relationship between agonists and antagonists during voluntary movements is altered in patients with spasticity including children with CP, and depressed spinal inhibition of sensory pathways has been noted in the majority of the studies in the past [12,13]. However, there are still some research studies that refute this theory [14].

Using non-invasive electrical and magnetic neurostimulation, alpha motoneuron inputs can be manipulated and such research may provide insight into the roles of particular human brain networks. The H-reflex is a well-studied and highly reproduced neurophysiological assessment of the proprioceptive sensory pathways that activate spinal motoneurons. It is a response that examines the overall reﬂex arc, including the Ia sensory aﬀerent strength and the spinal motoneuron excitability state. It is often regarded as the electrical equivalent of the spinal stretch reflex. An electrical stimulation test pulse is administered to a peripheral nerve to elicit the H-reflex. This activates Ia afferent axons from muscle spindles, which then stimulate alpha motoneurons, resulting in quantifiable muscle activity. Although H-reﬂexes are usually measured at rest, there is a considerable amount of evidence suggesting that these responses are dependent on the task performed and the context [15-17]. Moreover, not only active movement but also vibratory stimulation has been found to have a profound neuromodulatory influence on the H-reflex. The mechanisms, however, remain debatable. Proposed explanations for the H-reflex depression due to vibratory stimulation are diverse. Presynaptic inhibition, homosynaptic post-activation depression, vibration-induced reduced intrinsic motoneuron excitability, and the role of suprapinal mechanisms have been described in different research studies [17-21]. This vibration-induced H-reflex modulation has been reported to be impaired in spastic disorders [22]. 

Changes in the H-reflex during vibration and at the onset of voluntary movements reflect the function at the level of the spinal cord and the tonic descending influences which aid in reciprocal inhibition. The supraspinal component of the response can be tested by observing the changes in H-reflex before the onset of voluntary movements. In the current study, we sought to determine the neuronal mechanism involved, by analyzing H-reflex variations during voluntary movements and in response to vibratory stimulation. The study aimed to determine the components of the response which are affected in children with CP. Determining the involvement of reciprocal inhibition in spasticity can have important clinical implications in planning the treatment of the condition. Many researchers have also reported vibration as one of the antispasticity managements in CP patients [23]. Its potential use as an intervention in patients with increased spinal reflex excitability needs to be investigated. Further insight can be provided by the relative involvement of the spinal cord and supraspinal circuits, which may allow us to estimate the possible efficacy of different therapeutic approaches used to treat spasticity.

## Materials and methods

Twelve children with spastic CP (10 subjects were diplegic, 1 hemiplegic, and 1 quadriplegic; 10 males, 2 females) in the age group of 10-16 years were recruited by the homogeneous purposive sampling method. Children visiting the pediatric outpatient department at the institute, diagnosed with spastic CP, were included in the study group. The sample size calculation was done based on the difference in the percentages (increment/decrement) in the mean Soleus H-responses among cases and controls (effect size) from the previous similar study, with a power of 80% and 1.96 as the level of statistical significance [13]. Fifteen children with spastic CP were planned to be included based on the calculation. However, three children could not complete/be incorporated into the study owing to poor cooperation/learning disabilities; hence, 12 children constituted the study group. The control group comprised 15 age-matched healthy non-disabled children.

The study was conducted in the Neurophysiology Laboratory, Department of Physiology, All India Institute of Medical Sciences (AIIMS) Gorakhpur. Prior approval for conducting the study was obtained from the institutional ethics committee (Institutional Human Ethics Committee (IHEC), All India Institute of Medical Sciences, Gorakhpur, India, Reference number: IHEC/AIIMS-GKP/BMR/60/2022). It was a case-control study. Informed written consent from the parents and filled-out minor assent forms, where appropriate, were obtained from the children before the test. Children with a learning disability, seizures, previous selective dorsal rhizotomy, excessive lengthening of the Achilles tendon, or those on antispasticity drugs were excluded from the study.

H-reflex was recorded in the children at rest, during dorsiflexion, and during vibratory stimulation. All the children were tested, with periods of rest in between the trials. The children who could not complete the experiments on the scheduled day were called on a separate day for the rest of the trials. All the children had normal cognitive functioning and had a fair ability to perform the movements of the foot (dorsiflexion and plantar flexion) voluntarily.

Soleus H-reflex at rest

H-reflex was recorded on Neuro-MEPω electromyography and EP Digital Neurophysiological System software (M/S Neurosoft Ltd, Ivanovo, Russia) in Neurophysiology Laboratory, AIIMS, Gorakhpur. 

**Figure 1:**
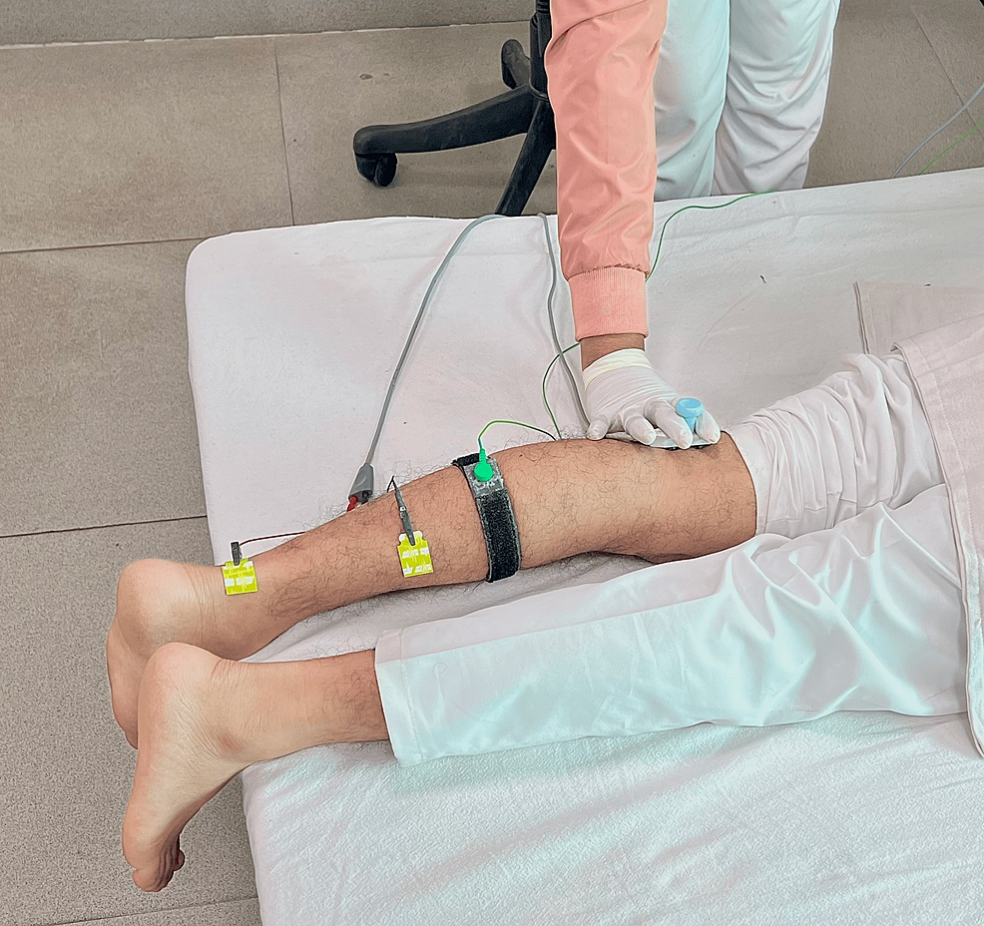
H-reflex recording procedure in a 16-year-old healthy male child at rest

The patient lay prone with leg and thigh firmly supported. The feet hung freely with the dorsum perpendicular to the tibia. Skin temperature was maintained at 32-33°C. Before recording the responses, the skin was cleaned with gauze moistened with methylated spirit. Surface adhesive disposable electrodes were used. An active surface electrode was placed at the distal edge of the calf muscle and a reference electrode on the Achilles tendon. The electrode impedance was kept below 10 kOhm. Stimulation was provided by the Neuro-MEP electrical stimulator at the popliteal fossa (square pulse of 1 ms duration and at a frequency of 0.5 Hertz) with the cathode proximally (Figure 1). The intensity of stimulation was increased from a subliminal level (in 1 mA increments) until an H-reflex without an M response was recorded. The H-reflex was identified as a triphasic wave with a small initial positive deflection followed by a larger negative one. The largest amplitude H-response observed was designated as the Hmax. The stimulus intensity was then further increased. A maximum or near-maximum M-response was then obtained depending on the patient's cooperation. The largest amplitude M-response obtained was designated as the Mmax. The stimulation and recording settings of the procedure were according to the study by Braddom and Johnson [24].

Soleus H-reflex with dorsiflexion

The child was instructed to dorsiflex the foot and maintain the position until the trial was completed. It was ensured by one of the experimenters that the child performed the movement correctly. Surface EMG was recorded for the tibialis anterior muscle during the test to ensure efficient contractions during the trial. The gain was 200 uv/division, sweep speed of 5 ms/division, and signals were ﬁltered by band-pass of 5 Hz-1 kHz.

The EMG signal crosstalk was negligible during all experiments. It was checked by recording the relative amplitudes of the M wave from surface electrodes on the tibialis anterior muscle after delivering sub-maximal/maximal electrical stimulation to the gastrocnemius soleus.

Soleus H-reflex during vibration

After a period of rest, H-reflex was tested for vibratory stimulation (Wahl vibrator, Model 4196, Sterling, Ill) over the Achilles tendon (frequency 60 Hz), proximal to the reference electrode. As the testing stimulation was started, the vibrator was firmly pressed onto the tendon a few cm above the ankle joint. Data was collected during the constant application of vibration.

Five H-reflex trials were recorded for each experiment, to ensure reproducibility, and the electrophysiological parameters were recorded for further analysis. 

The maximum amplitudes of the H-reflex and the M-wave were measured as the difference between the peaks of the positive and negative deflections. All H-reﬂex measurements were normalized to M max. The Hmax-to-Mmax ratio was calculated by dividing the maximum amplitudes of the H-reflex by that of the M-wave.

Statistical analysis included computation of mean, standard deviation, median, and interquartile range for the variables. The Wilcoxon signed-rank test and the two-sample Kolmogorov-Smirnov (KS) test were used for comparing and analyzing the data. p-value <0.05 was considered as statistically significant. The analyses were performed using IBM SPSS Statistics for Windows, Version 28 (Released 2021; IBM Corp., Armonk, New York, United States).

## Results

**Figure 2:**
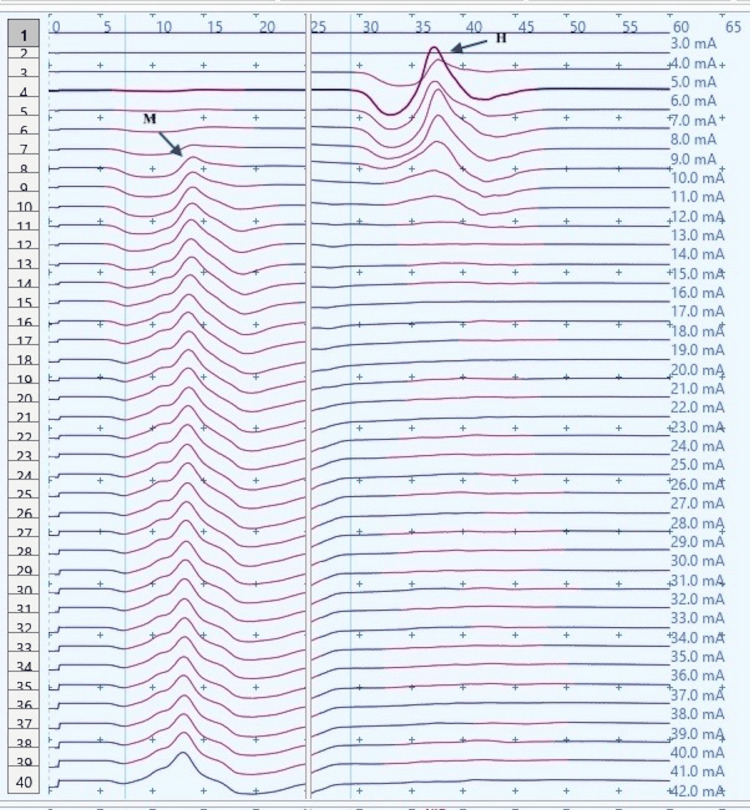
H-reflex traces in a control male child at rest (sweep speed 5 ms/division, sensitivity: 20 mV/division (M response), 4 mV /division (H response)) ms: millisecond; mV: millivolt; H: H response traces; M: M response traces (black arrows).

**Figure 3:**
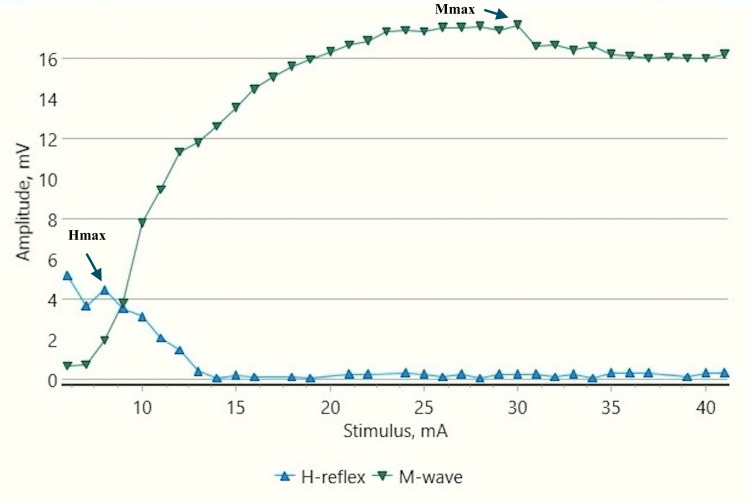
Recruitment curve for H and M-response amplitudes (mV) with stimulus intensity increments (mA) in a control male child at rest mV: millivolt; mA: milliampere; Hmax/Mmax: 29.6%, Hmax: 5.24 mv, Mmax: 17.7 mv (black arrows).

**Figure 4:**
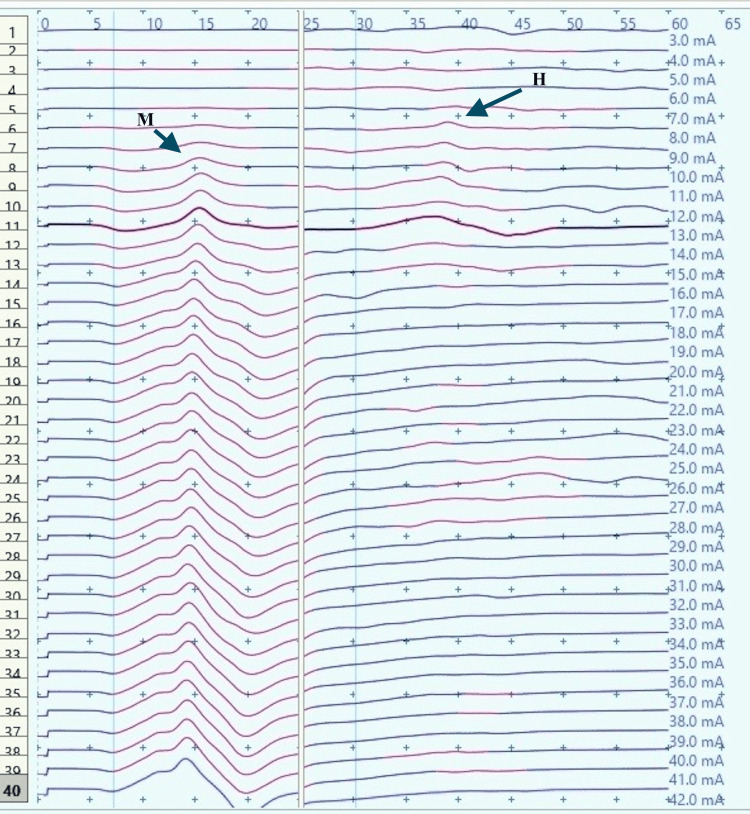
H-reflex traces in a control male child with dorsiflexion (sweep speed 5 ms/division, sensitivity: 20 mV/division (M response), 4 mV /division (H response)) ms: millisecond; mV: millivolt; H: H response traces; M: M response traces (black arrows).

**Figure 5:**
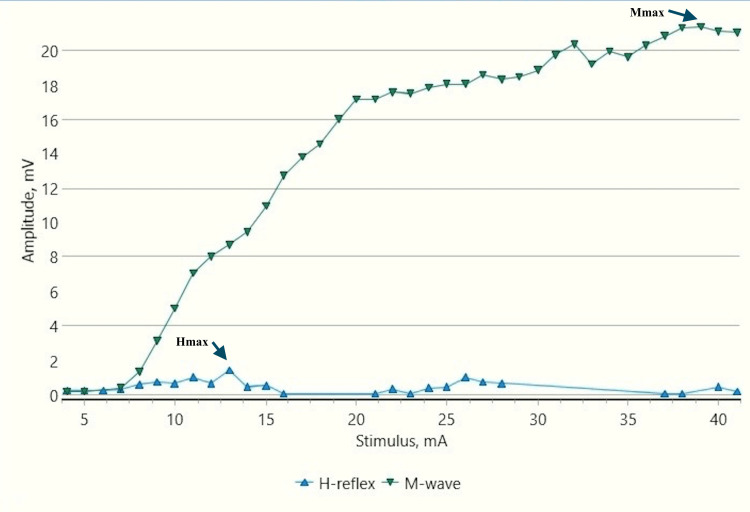
Recruitment curve for H and M-response amplitudes (mV) with stimulus intensity increments (mA) in a control male child during dorsiflexion A substantial decrease in Hmax and Hmax/Mmax ratio by about 70% and 77% respectively, from the resting values (Figure 3) can be noted. mA: milliampere; mV: millivolt; Hmax/Mmax: 6.8.%, Hmax: 1.46 mv, Mmax: 21.4 mv (black arrows).

**Figure 6:**
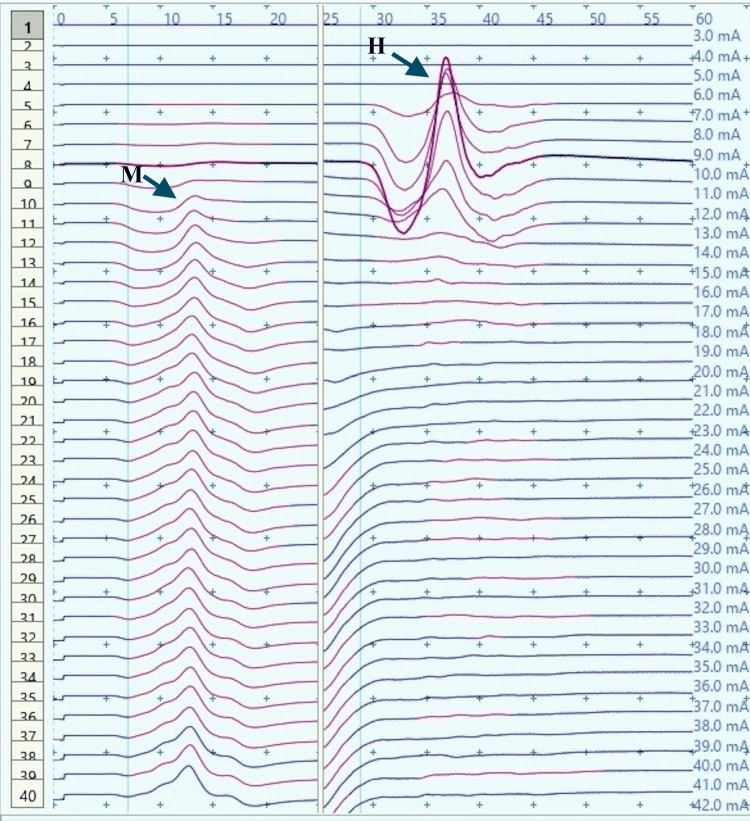
H-reflex traces in a male child with CP during rest (sweep speed 5 ms/division, sensitivity: 20 mV/division (M response), 4 mV /division (H response)) CP: cerebral palsy; ms: millisecond; mV: millivolt; H: H response traces; M: M response traces; (black arrows).

**Figure 7:**
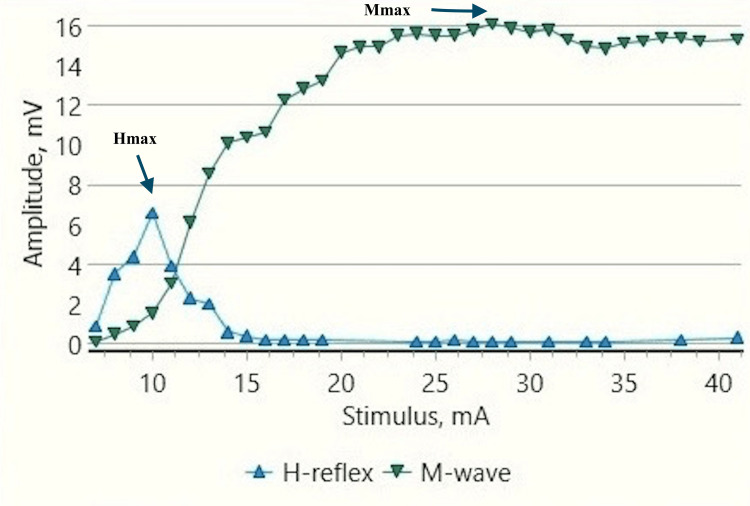
Recruitment curve for H and M-response amplitudes (mV) with stimulus intensity increments (mA) in a male child with CP at rest CP: Cerebral palsy; mA: milliampere; mV: millivolt; Hmax/Mmax: 40.99%, Hmax: 6.6 mv, Mmax: 16.1 mv (black arrows).

**Figure 8:**
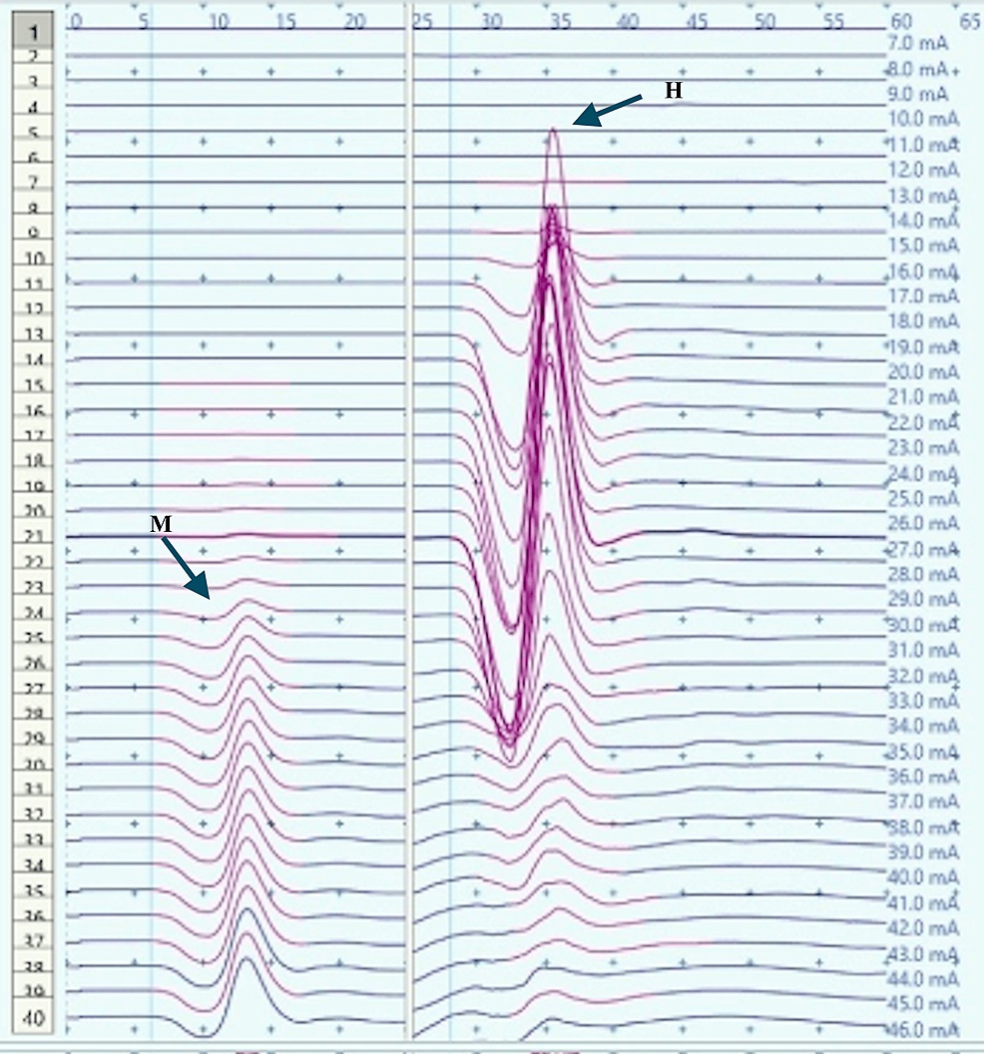
H-reflex traces in a male child with CP during dorsiflexion (sweep speed 5 ms/division, sensitivity: 20 mV/division (M response), 4 mV /division (H response)) CP: Cerebral palsy; ms: millisecond; mV: millivolt; H: H response traces; M: M response traces (black arrows).

**Figure 9:**
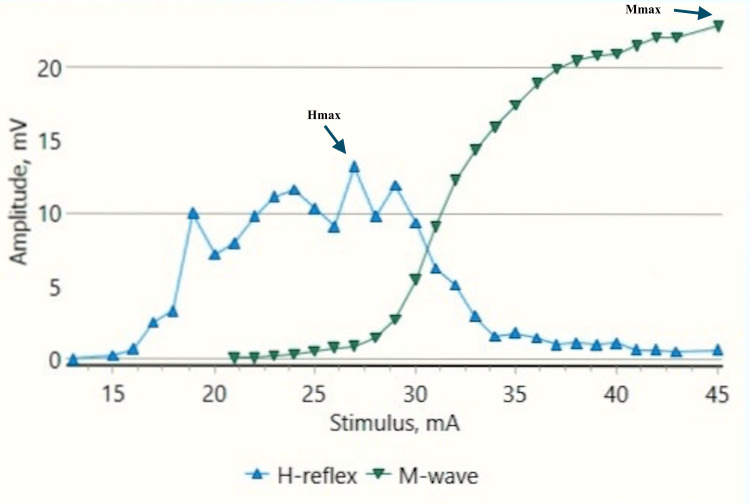
Recruitment curve for H and M-response amplitudes (mV) with stimulus intensity increments (mA) in a male child with CP during dorsiflexion A substantial increase in Hmax and Hmax to Mmax ratio from the resting values (Figure 7) can be noted. CP: Cerebral palsy; mA: milliampere, mV: millivolt; Hmax/Mmax: 57.5.%, Hmax: 13.1 mv, Mmax: 22.9 mv (black arrows).

**Figure 10:**
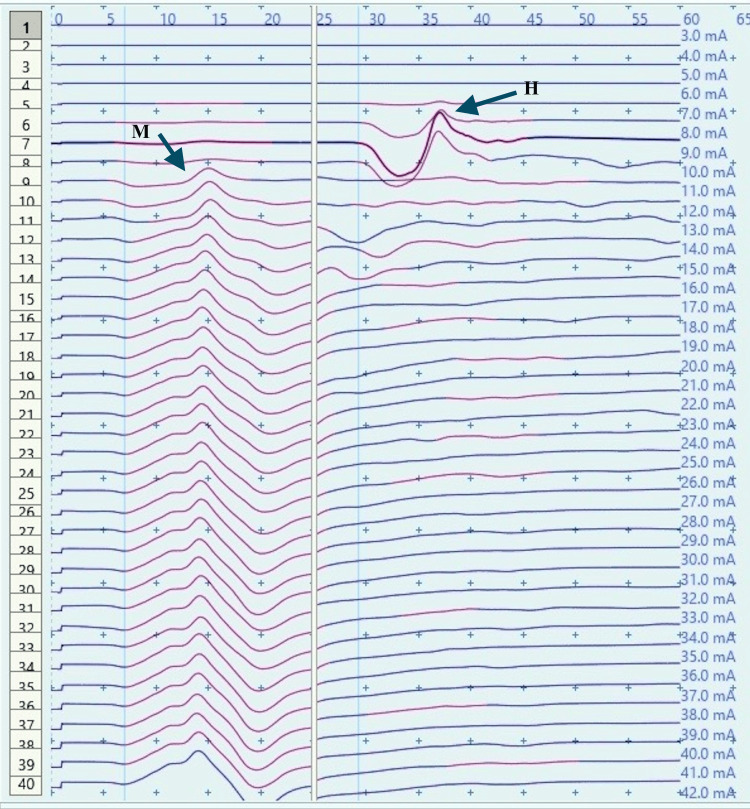
H-reflex traces in a control male child during vibration (sweep speed 5 ms/division, sensitivity: 20 mV/division (M response), 4 mV /division (H response)) ms: millisecond; mV: millivolt; H: H response traces; M: M response traces (black arrows).

**Figure 11:**
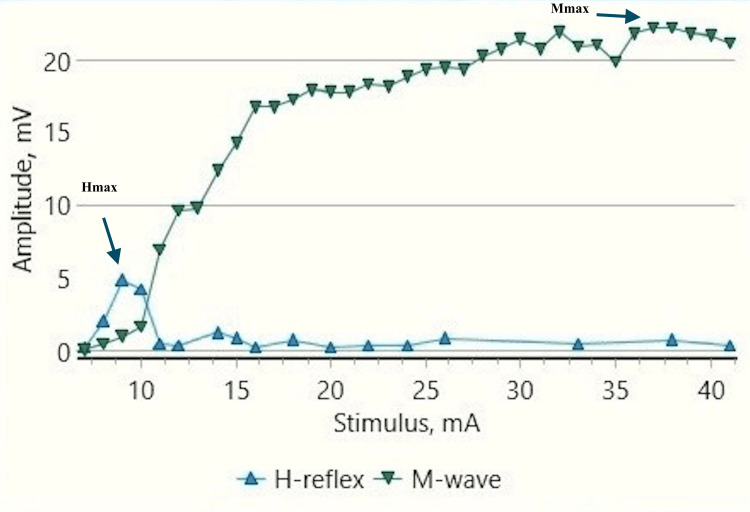
Recruitment curve for H and M-response amplitudes (mV) with stimulus intensity increments (mA) in a control male child during vibration A mild decrease in Hmax amplitudes and Hmax/Mmax can be noted as compared to resting values (Figure 3). mA: milliampere; mV: millivolt; Hmax/Mmax: 21.9%, Hmax: 4.84 mv , Mmax: 22.1 mv (black arrows).

**Figure 12:**
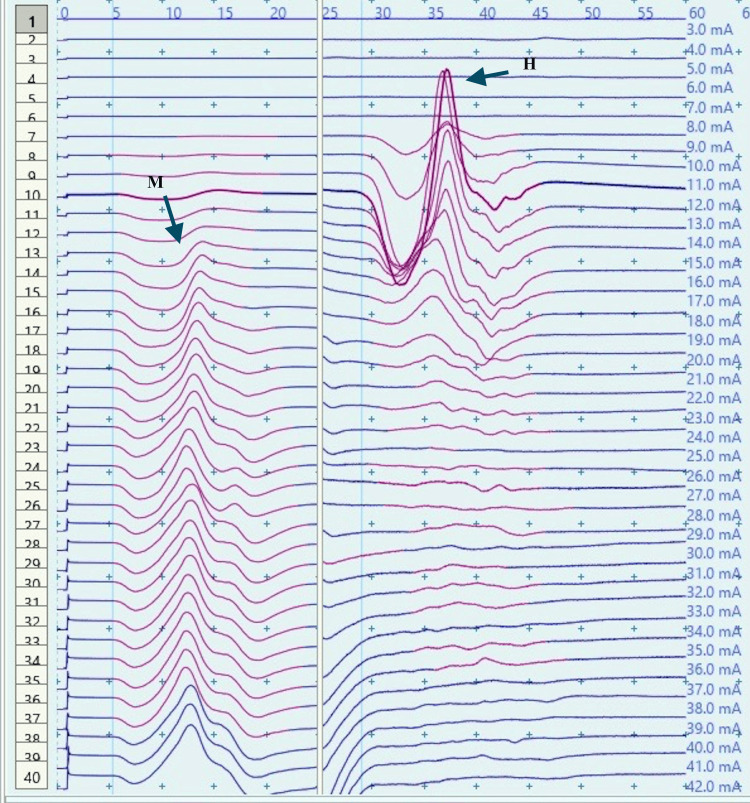
H-reflex traces in a male child with CP during vibration (sweep speed 5 ms/division, sensitivity: 20 mV/division (M response), 4 mV /division (H response)) CP: cerebral palsy; ms: millisecond; mV: millivolt; H: H response traces; M: M response traces (black arrows).

**Figure 13:**
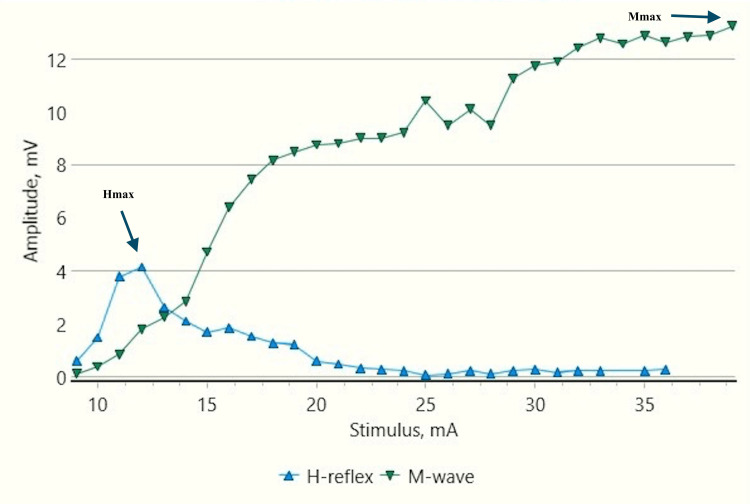
Recruitment curve for H and M-response amplitudes (mV) with stimulus intensity increments (mA) in a male child with CP during vibration A decrease in Hmax amplitudes and Hmax/Mmax as compared to the resting values can be noted (Figure 7). CP: Cerebral palsy; mA: milliampere; mV: millivolt; Hmax/Mmax: 29.56%, Hmax: 4.11mv, Mmax: 13.9 mv (black arrows).

The H-response was recorded in 12 children with spastic CP (mean age ± SD: 13.87 ± 1.8 years) and 15 controls (mean age ± SD: 12.93 ± 2.1 years). A total of 22 H-reflex responses were obtained in the children with CP (from the bilateral lower extremities in 10 children while unilateral recording in two children). The responses were recorded at rest, during dorsiflexion, and during vibratory stimulation in each child (Figures 2-13). Median and interquartile ranges (IQR) were calculated for Hmax amplitudes and Hmax to Mmax ratio, for all the experiments performed. The control group included a total of 30 H-reflex responses from the bilateral lower extremities. Each response included the recording of H-reflex at rest, during dorsiflexion, and during vibratory stimulation. The data was compared among the controls and the cases, for the experiments performed i.e. during rest, during dorsiflexion, and during vibratory stimulation by the Wilcoxon signed-rank test. The recruitment curves (which depicted the distribution of H-response amplitudes with stimulus intensity increment value) for dorsiflexion were compared among controls and cases by the two-sample KS test. A similar comparison was performed among the groups for recruitment curves obtained during vibration. 

H reflex response in children during dorsiflexion

Control Children

H-response in control children demonstrated a substantial decrease in the Hmax and Hmax to Mmax ratio during dorsiflexion as compared to those obtained at rest (Figures 2-5). The Hmax value (median, IQR) (5.23 mV, 0.69) was found to be decreased by about 62% after dorsiflexion (2.08 mV, 0.3). The decrease in the amplitudes was statistically significant with p=0.00006 (Wilcoxon signed-rank test). Similarly, a decrease in the Hmax-to-Mmax ratio (median, IQR) was noted which was 28%, 2 at rest while 12%, 1 during dorsiflexion. The drop in the ratio was also found to be statistically significant (p=0.0007) (Table 1).

**Table 1:** H-response (Hmax and Hmax/Mmax %) compared during rest and dorsiflexion in controls and cases (Wilcoxon signed-rank test). mV: millivolt; IQR: interquartile range

	Hmax (mV) Median (IQR)		Hmax/Mmax % Median (IQR)	
	Controls	Cases	Controls	Cases
Rest	5.24 (0.69)	6.8 (1)	28 (2)	32 (5)
Dorsiflexion	2 (0.3)	8 (2.3)	12 (1)	36 (5)
p value	0.00006	0.018	0.0007	0.01

Children With CP

H-response recording during dorsiflexion in the cases provided contrasting results as compared to those in controls (Figures 5, 9) with an increase in Hmax from the resting value (6.8 mV, 1) to 8 mV, 2.3 during dorsiflexion (Figures 7, 9). This increment of about 15 % was found to be statistically significant with a p-value of 0.018 (Wilcoxon signed-rank test). A similar increase was observed for the Hmax/Mmax ratio which was 32 %, 5 at rest while 36 %, and 5 during dorsiflexion (statistically significant, with p value=0.01) (Table 1).

H reflex response in children during vibration

Control Children

Vibratory stimulation produced a decrease in H-response amplitude in control children (Figures 10, 11) from the resting values (Figures 2, 3). About 24% decrease (5.23 mV, 0.69 to 4 mV, 1.34) was found in the median values obtained in the experiments during vibration while Hmax/Mmax (28%, 2 at rest while 22%, 1 during vibration) showed a decrease of about 21% (p-value of 0.012 and 0.0007, respectively) (Table 2).

**Table 2:** H-response (Hmax and Hmax/Mmax %) compared during rest and vibratory stimulation in controls and cases (Wilcoxon signed-rank test). mV: millivolt; IQR: interquartile range

	Hmax (mV) Median (IQR)		Hmax/Mmax % Median (IQR)	
	Controls	Cases	Controls	Cases
Rest	5.24 (0.69)	6.8 (1)	28 (2.0)	32 (5.0)
Vibration	4.0 (1.34)	5.8 (0.6)	22 (1.0)	27 (2.0)
p value	0.012	0.003	0.0007	0.0033

Children With CP

In children with CP, a similar decreasing trend (Figures 12, 13) was found in Hmax amplitudes and the H max/Mmax ratio recorded during vibration from the resting values (Figures 6, 7). The decrease was about 15 % (6.8 mV, 1 to 5.8 mV, 0.6) (p-value: 0.003) and 16% respectively (32%, 5 to 27%, 2) (p-value: 0.0033) from the resting values (Table 2).

**Figure 14:**
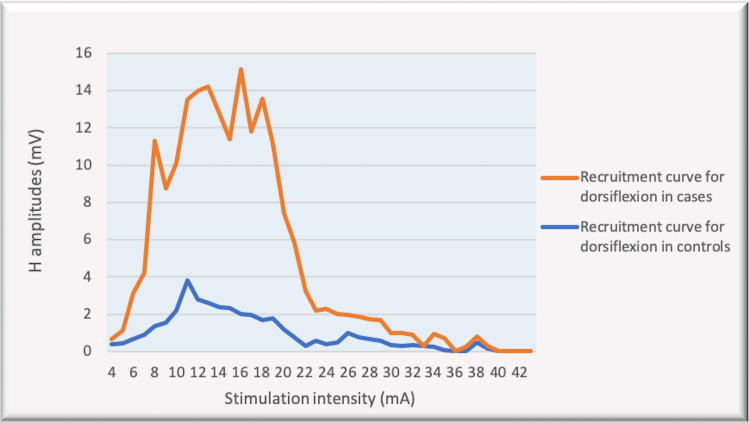
Comparison of the recruitment curves depicting distribution of average H amplitudes (mV) with stimulation intensity (mA) recorded during dorsiflexion among controls and cases. mV: millivolt; mA: milliampere

**Figure 15:**
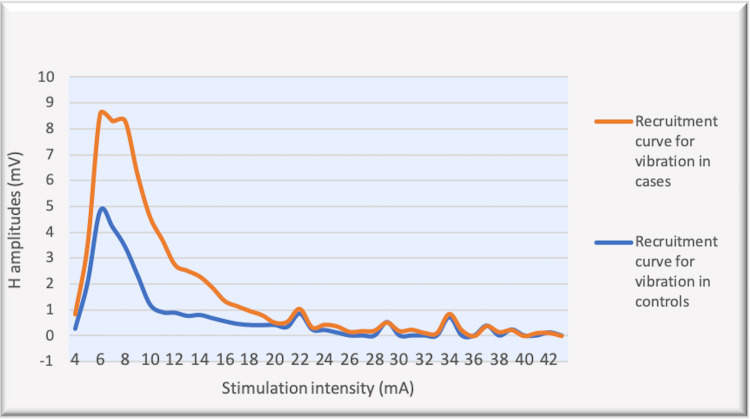
Comparison of the recruitment curves depicting distribution of average H amplitudes (mV) with stimulation intensity (mA) recorded during vibration among controls and cases. mV: millivolt; mA: milliampere

The recruitment curves for the distribution of average H amplitudes with stimulation intensity recorded during dorsiflexion and vibration experiments were compared (Figures 14, 15). Vibration depicted a nearly similar trend with stimulation increment in cases, as compared to controls, however, with a larger curve. Dorsiflexion, on the other hand, demonstrates a considerable difference in the curves for controls and cases. Children with CP showed a greater and sharper increase in average H amplitude with respect to stimulation intensity as compared to controls. The differences in the distribution were found to be statistically significant by the two-sample KS test (p<0.0001).

## Discussion

CP has been associated with the presence of a signiﬁcant coactivation of muscles [25,26]. Muscle co-activation has been described as the coincident contraction of agonist and antagonist muscles around a joint. In this regard, Ia reciprocal inhibition has been suggested as one of the important mechanisms involved in the coordination of antagonist muscles. However, previous experiments performed at rest have reported no change in reciprocal inhibition in individuals with CP as compared to healthy controls [27]. This can be explained based on the fact that the modulation of the spinal motor circuitries during motor activities is not similar to during rest [28]. Leonard et al. reported that the reciprocal inhibition was increased during static agonist contraction, in healthy subjects and reduced in CP, while it was similar when measured at rest [29]. A decrease in the size of an unconditioned Soleus H reflex has been reported to be associated with the dorsiflexion of the foot in normal adults [30]. Present experiments in this study have also found substantial inhibition in normal children during voluntary movement in the form of dorsiflexion of the foot (p<0.0001 and p<0.001 for Hmax and Hmax/Max respectively) (Table 1, Figures 2-5). On the other hand, greater Soleus H-reflex response obtained in children with CP with the voluntary movement of the antagonist muscle during dorsiflexion supports the presence of impaired reciprocal inhibition in the group (Table 1, Figures 6-9) (p<0.05 for both Hmax and Hmax/Mmax). A substantial increase in the Hmax and Hmax/Mmax values in children with CP during dorsiflexion was a prominent finding in the present study. Although the majority of the studies have confirmed the presence of a larger Soleus H response during dorsiflexion in adults with CP, a few similar studies performed in children support the present findings [13]. A large stretch reflex/M-response area/larger Hmax to Max ratio in CP at rest as reported in the present study has also been interestingly reported in children with spastic CP at rest which is attributable to enhanced stretch reflex excitability [31,32]. The degree of spasticity has been associated with the amount of Ia inhibition. A significant increase in la inhibition was found in the less spastic patient. A considerable increase in H response amplitudes during dorsiflexion found in our study could be attributed to the degree of spasticity among the children.

In children who suffer an early brain insult, reorganization of supraspinal input and impaired motor maturation are involved in the development of spasticity and impaired reciprocal inhibition, unlike the pathology in adults whose motor system is developed at the time of injury. The study involving how maturation affects the pathophysiology of spasticity and impaired reciprocal inhibition is still in its early stages. If the involvement of age-related variations in the underlying neural pathways is ascertained for the varying motor deficits in adults and children with early brain injury, it would be helpful for further understanding.

Soleus H-reflex responses after vibratory stimulation test the integrity of the spinal cord circuitry. H-reflex depression due to vibratory stimulation has been most commonly explained by presynaptic inhibition in which Ia afferents are blocked when they stimulate GABAergic interneurons. This momentarily prevents further stimulation [17-19]. Homosynaptic post-activation depression is another suggested mechanism that is due to a decrease in the probability of neurotransmitter release at the Ia alpha motoneuron synapse as a result of repeated activation of Ia afferents during vibration [20]. Recently, reduced intrinsic motoneuron excitability due to vibration has also been suggested, which is more responsible for the suppression of H-reflex than either homosynaptic post-activation depression or presynaptic inhibition [21].

Vibration has been studied extensively for modulation of spinal motoneuronal excitability in individuals with spinal cord injury and significant inhibition of the H-reflex after vibration onset (up to 9%) has been reported [33]. Similar studies investigating H-reflex response during vibratory stimulation in children with CP are relatively fewer. Leonard et al. have conducted experiments in children with CP and reported a decrease in the Soleus H-response amplitudes after vibratory stimulation [13]. The results from our study also demonstrated a reduction in the Soleus H response in controls (p<0.05 and p<0.001 respectively) as well as in children with CP (p<0.05 for both Hmax and Hmax/Max %) (Table 2). However, the degree of inhibition among the children with CP was smaller as compared to the controls (Figures 14, 15), (p<0.0001) (two-sample KS test). Therefore, it is difficult to conclude that the Ia inhibitory pathway and/or presynaptic inhibition pathway were operating in children with CP unaltered and that the impairment at the level of segmental circuitry cannot be excluded in CP. 

Limitations

If H-response alterations could have been temporally compared with the commencement of voluntary movement, a supraspinal origin of movement-induced modulation of reciprocal inhibition would have been observed. In our experiment, we recorded the H-response changes after the onset of dorsiflexion; hence, we could not determine the supraspinal influences. Second, the level of voluntary contractions during dorsiflexion was assessed by EMG recording during the trials; however, quantitative feedback to maintain the constant level of contraction could not be provided. Third, the experiments could have been performed with a larger sample size which would have further strengthened the results obtained.

## Conclusions

Children with CP have abnormal H-reflex patterns with larger H-responses at rest which accentuates during dorsiflexion. The lack of H-reflex suppression during voluntary antagonist muscle activation suggests the presence of an impaired reciprocal inhibition mechanism in CP. Relatively modest H-response reduction by vibratory stimulation in children with CP provides equivocal evidence of vibratory modulation of H-reflex in CP. Further studies exploring the mechanisms underlying motor deficits and spinal reflex excitability in children with CP are warranted which, in turn, can have a significant role in planning the treatment of the condition.
